# International medical students – a survey of perceived challenges and established support services at medical faculties

**DOI:** 10.3205/zma000951

**Published:** 2015-02-11

**Authors:** D. Huhn, F. Junne, S. Zipfel, R. Duelli, F. Resch, W. Herzog, C. Nikendei

**Affiliations:** 1 University Hospital Heidelberg, Centre for Psychosocial Medicine, Department of General Internal Medicine and Psychosomatics, Heidelberg, Germany; 2 Eberhard-Karls-University Tubingen, Medical Clinic, Department of Psychosomatic Medicine and Psychotherapy, Tübingen, Germany; 3 Ruprecht-Karls-University of Heidelberg, Dean's Office of the Medical Faculty of Heidelberg, Heidelberg, Germany

**Keywords:** international medical students, migration, medical curriculum

## Abstract

**Introduction: **Medical students with a non-German background face several challenges during their studies. Besides support given by foreign student offices further specific projects for international students have been developed and are offered by medical faculties. However, so far, neither a systematic survey of the faculties’ perceived problems nor of the offered support exists.

**Method: **All study deaneries of medical faculties in Germany were contacted between April and October 2013 and asked for their participation in a telephone interview. Interview partners were asked about 1.) The percentage of non-German students at the medical faculty; 2.) The perceived difficulties and problems of foreign students; 3.) The offers for non-German students; and 4.) The specification of further possibilities of support. Given information was noted, frequencies counted and results interpreted via frequency analysis.

**Results: **Only 39% of the medical faculties could give detailed information about the percentage of non-German students. They reported an average share of 3.9% of students with an EU migration background and 4.9% with a non-EU background. Most frequently cited offers are student conducted tutorials, language courses and tandem-programs. The most frequently reported problem by far is the perceived lack of language skills of foreign students at the beginning of their studies. Suggested solutions are mainly the development of tutorials and the improvement of German medical terminology.

**Discussion: **Offers of support provided by medical faculties for foreign students vary greatly in type and extent. Support offered is seen to be insufficient in coping with the needs of the international students in many cases. Hence, a better coverage of international students as well as further research efforts to the specific needs and the effectiveness of applied interventions seem to be essential.

## Zusammenfassung

**Einleitung:** Internationale Medizinstudierende sind im Rahmen ihres Studiums in Deutschland mit einer Reihe von Herausforderungen konfrontiert. Neben der Unterstützung durch die zuständigen Akademischen Auslandsämter werden zunehmend auch spezifische Angebote für internationale Studierende durch die Medizinischen Fakultäten selbst entwickelt und angeboten. Eine systematische Übersicht über die Art und Verbreitung solcher Angebote sowie die von Seiten der Fakultäten wahrgenommenen Problemfelder für internationale Studierende existiert bislang nicht.

**Methode: **Alle Studiendekanate der Medizinischen Fakultäten Deutschlands wurden zwischen April und Oktober 2013 kontaktiert und um Teilnahme an einem Telefoninterview gebeten. Die Interviewpartner wurden zu folgenden Sachverhalten befragt: 1.) Anteil internationaler Studierender an der Medizinischen Fakultät; 2.) Wahrgenommene Schwierigkeiten und Probleme internationaler Studierender; 3.) Angebot für internationale Studierende; 4.) Spezifizierung des weiteren Bedarfs an Unterstützungsmöglichkeiten. Die Angaben wurden notiert, Häufigkeiten im Anschluss ausgezählt und die Ergebnisse frequenzanalytisch ausgewertet.

**Ergebnisse: **39% der Medizinischen Fakultäten konnten detaillierte Auskunft über den Anteil internationaler Studierender geben. Von diesen Fakultäten wurde ein durchschnittlicher Anteil von 3,9% EU- und 4,9% Nicht-EU-Ausländer angegeben. Die häufigsten genannten Angebote sind durch andere Studierende geleitete Tutorien, Sprachkurse und Tandem-Programme. Das mit Abstand am häufigsten berichtete Problem ist laut den Studiendekanaten das sprachliche Defizit der internationalen Studierenden zu Beginn des Studiums. Als Lösungsvorschläge werden hauptsächlich der Ausbau von Tutorien sowie die Verbesserung der fachsprachlichen Deutschkenntnisse genannt.

**Diskussion: **An den Medizinischen Fakultäten bestehen in Art und Umfang sehr unterschiedliche Unterstützungsangebote für internationale Studierende. In vielen Fällen wird das Angebot als unzureichend erlebt, um den Bedürfnissen der internationalen Studierenden gerecht zu werden. Eine bessere Erfassung der internationalen Studierenden sowie verstärkte Forschungsbemühungen zum spezifischen Bedarf sowie zur Wirksamkeit der angewandten Interventionen erscheinen in diesem Zusammenhang dringend notwendig.

## Introduction

More than 2000 international students enroll for medicine at a German university each year. This corresponds to a share of about 15% of all medical students [1]. Students who have completed their previous educational career in another country and only enter Germany for the purpose of further university education are referred to as "international students". The term "foreign students" refers to students who have lived in Germany before taking up their university education and have also acquired their university entrance qualification here, however, are not in possession of German citizenship [2], [3]. Furthermore, the differentiation in "non-mobile foreign students" and "foreign students" is also prominent. The former have a German university entrance qualification, which they have acquired in Germany or at a German school abroad; latter have acquired their university entrance qualification abroad and have enrolled at a German university after recognition of their university degree [3]. A further distinction is made between nationals of European Union Member States and other international citizens [1]. This is of particular relevance since the modernization of European educational structures in the wake of the Bologna Process has facilitated the enrollment of EU citizens in Universities abroad within the European Union [http://www.eua.be/eua-work-and-policy-area/building-the-european-higher-education-area/bologna-basics.aspx retrieved 18.12.2013], [4]. The foundation "Stiftung für Hochschulzulassung" (SfH, eng.: University Admissions Foundation), functioning as the central body for the allocation of university places in medicine in Germany, does not differentiate between non-German EU citizens and German citizens. In consequence, equal opportunities in the allocation of medical study places are given [1]. For non-EU citizens, however, the approval for the enrollment in the study of medicine is under quota regulation. The SfH is able to allocate up to 5% of available university places in medicine to non-EU citizens. Hence, up to 5% of places are awarded to non-EU foreigners, after respective assessment tests and proof of sufficient language skills, nationwide [1].

These figures show that international medical students represent a group which should be given special attention in terms of social integration and in respect to study-related performance demands. However, research efforts on this topic are still described as inadequate, in particular, in the German language area [5], [6], [7]. International studies have shown that international medical students are more frequently prone to report personal stress [8], reduced quality of life [6], [9], lack of support [10], [6] as well as lack of social contacts [6] [11] in the course of their studies and also prove to hold higher dropout rates than domestic medical students [12], [11] or come to attain their degrees only after a significantly higher number of semesters [13]. Moreover, exams also appear to be a greater challenge for international students [14], [15], a fact which is reflected in both poorer results in written, oral and practical clinical examinations [16], [17], [18], [19], [20], [21], [22]. In a separate study, we were also able to show for the German language area that students with a non-European immigrant background achieved significantly lower results in written exams during preclinical semesters as well as in the oral state examination and also took the state examination after a significantly higher amount of semesters compared to their fellow German students [23].

The International Relations Office (dt.: Akademisches Auslandsamt) [https://www.daad.de/deutschland/in-deutschland/hochschule/de/9147-der-erste-ansprechpartner/ retrieved 18.12.2013], sometimes referred to as the "International Office" or "Office for International Affairs", is usually a first point of contact for international students from all disciplines at German universities. Here, already admitted international students and in some cases also international applicants are able to find broad support in queries and issues that affect their university education as well as receiving required help regarding their private matters [https://www.daad.de/deutschland/in-deutschland/hochschule/de/9147-der-erste-ansprechpartner/ retrieved 18.12.2013]. However, the medical faculties themselves seem to be more and more aware of the international students’ specific situation. Nevertheless, so far no systematic overview of the support services provided by medical faculties exists to our knowledge.

The aim of the present study was to obtain a systematic overview of the medical faculties’ assessment of the situation of international students by means of a nationwide telephone survey covering the following aspects: perceived problem areas, possible solution proposals and existing support services.

## Method

### Objective of the study and study design

All study deaneries of the 36 medical faculties of the Federal Republic of Germany were contacted via telephone between April and October 2013 and asked to participate in the survey. The aim was to ascertain how the situation of international medical students is evaluated by the representatives of the study deaneries. The contacted heads of the deaneries were able to decide whether they themselves wanted to partake in the survey as interviewees or in turn wanted to refer the interviewer to a competent colleague. Hence, a competent representative was interviewed for each faculty. All interviews were conducted exclusively by the first author of this paper (DH).

#### Interview guideline

A compact and clearly structured interview guideline was designed for the telephone interview [24] and divided into the following sections:

1. Proportion of international students at the medical faculty, differentiated according to EU and non-EU foreigners
2. Perceived difficulties and problems of international students
3. Existing support services for international students
4. Perceived solution possibilities to meet the needs for support

Respectively, the question on the proportion of international students is quantitative; all others are of a qualitative nature. Response categories were not predetermined at any time.

#### Interview implementation

In the course of the interview, open questions were read out to the interviewees to which they could answer freely. The interviewer could also provide expanding and clarifying questions to gain additional information. Paper and pen notes were taken during the interview. The telephone interviews lasted between 10 and 30 minutes. The interviewees were also able to submit additional information via e-mail if the required data was not available during the interview call.

#### Categorization of the statements

The documented responses were later examined regarding to similarities and differences during evaluation. The evaluation was carried out by two independent raters not participating in the survey. Discrepancies were discussed in the process. Although the evaluation was based on the methodology of content analysis (e.g. Grounded Theory), the focus was set on the categorical assignment of clear terminological concepts as defined in frequency analysis [25] rather than on a content-analytical approach (i.e. analysis of content meaning). Strongly converging answers were assigned to the same category.

## Results

### Proportion of faculties participating in the survey

A competent representative of the dean of studies office of each of the in total 36 existing medical faculties in Germany participated in this survey. The competent representative was either the respective head of the deanery himself or alternatively a competent representative designated by the head who could provide information on international students at the faculty or a person who held a position of responsibility in this area. Accordingly, deans, managers and staff of study deaneries participated in this survey, as well as "diversity officers" working with international students on a daily basis. The respondents' answers were based - with the exception of data on the proportion of international students - exclusively on their subjective assessment. 100% of the medical faculties participated in this survey and 36% of Interviewees made use of the option of submitting further information via e-mail.

#### Proportion of international students 

42% of the medical faculties could not give any indication in regard to the question on the share of international medical students at their faculty differentiated according to EU and non-EU foreigners. These medical faculties had neither appropriate statistics available, nor were they able to ascertain how such information could be obtained. Further 19% of the faculties could only report estimated figures, while 39% of the medical faculties were able to present detailed information on the percentage of EU and non-EU foreigners in relation to the total number of their medical students. Averaged across these faculties, EU-foreigners made up 3.9% of the total enrolled students and a share of 4.9% of non-EU foreigners was reported respectively (see table 1 (Tab. 1)).

#### Perceived difficulties and problems of international students

For 83% of the interviewed representatives, difficulties with the German language posed the biggest challenge to international students. Languages skills were mostly perceived to be particularly low at the beginning of the international students’ enrollment, which consequently were seen to make it difficult for students to meet the full demands of the medical courses. 36% of the faculties reported to feel challenged with the integration of the international students in the group of all other students. However, the interviewees also stressed that international students had difficulties, in turn, in utilizing provided support services as they often grouped themselves with peers of a similar cultural background. 28% of the interviewees stated that often intercultural differences were also a decisive factor when regarding the problems of international students. Accordingly, in many cases international students were accustomed to different methods of education, study and work and need to adjust to the given circumstances in the first few months. A further problem, as was reported by 28% of the representatives, is that distressed international students would often fail to seek help or would request support far too late. 28% of respondents addressed the often precarious financial situation of international students as a further difficulty. In order to finance their studies, many would have to work while studying leading to lower capacities for revising and learning the syllabus. 11% of the interviewed representatives saw a major difficulty in the lower level of training, to their perception, of many international students, in particular, in the natural sciences compared to their German fellow students. One faculty reported that the issue of discrimination in the context of courses through fellow students or lecturers proved to be a big problem for international students. Also one faculty indicated that many of the existing problems sprung from the immense social pressure (e.g. not wanting to disappoint the family, since they had made it possible to study abroad) that many international students were under (see table 2 (Tab. 2)). 

#### Services for International Medical Students

Student-led tutorials for preclinical courses were the most common specific offer provided to international medical students. 44% of medical faculties stated that they provided these during the first two semesters in the majority of cases. During these tutorials, students are given the possibility to revise lesson content, learn subject-specific knowledge and prepare for upcoming exams. 36% of medical faculties reported to offer specific language courses for international medical students with the aim to improve subject-specific vocabulary in German and Latin in particular. So called “tandem programs” were offered at 31% of the medical faculties. Here, a medical student in a higher semester is paired with an international medical student, who has just arrived in Germany, and supports him during the difficult start-up phase. 25% of the faculties provided freshmen-specific counseling services for international students in the context of introductory courses informing students about different contact points, giving an overview of the medical course content in general, and explaining the most important terms of German university life. Individualized support services for international students were offered by a total of 22% of medical faculties. Services reported here, spanned from co-called mentor programs, in which international students can seek advice form older students or even lecturers, over designated "diversity officers" to specific consultations hours for international students. Only 11% of the faculties offer additional examination perpetration courses to international students, in which examination related content is revised separately from other students. A recreational program tailored to the specific needs international students, including regular meetings, football tournaments etc. was reported by one faculty, which also permits international students the use of dictionaries during written examinations. Overall, 14% of medical faculties did not have a specific offer for their international students (see table 3 (Tab. 3)).

#### Perceived possible solutions

In respect to possible solutions, 19% of the representatives of medical faculties saw the further expansion of tutorials for international students, within which educational content and questions can be asked, as an opportunity for improvement. A further 19% of the interviewees were in favor of increasing efforts in teaching both German language skills as well as subject-specific knowledge (as in natural sciences) to international students before the commencement of their university courses in order to ensure that these students are able to meet the demands of medical courses from the start. 14% of respondents saw the establishment of specific medical German language skill courses during university education as a great way to support international students. 11% perceived the establishment of appropriate examination preparatory courses tailored to the specific needs of international students as an appropriate measure of improvement. 8% of the representatives saw better financial support for international students as a possible solution giving students the chance to fully concentrate on their studies. However, 8% of the interviewees highlighted that only an increase of the financial and / or human resources could provide a real improvement in the support services offered to international students in the long run. Again, 8% saw the more targeted choice of students in advance and the entailed greater selection of highly qualified applicants as a good possibility to promote the admission of international students with a particularly promising outcome. Likewise, training in intercultural skills, which would be made available for the entire student body, and in consequence benefit German as well as their fellow international students, was seen as a good opportunity for improvement. Further 8% of the representatives saw the increased development of individual counseling services for international students as a possible solution. Seven further aspects, seen as good improvement possibilities, were mentioned once each; a detailed list of these is given in table 4 (Tab. 4). Notwithstanding, 8% of the faculty representatives indicated that they deemed the support services, they were able to provide to international students, as sufficient.

## Discussion

To the authors' knowledge the present study is the first in which perceived problem areas and potential possibilities for the support of international medical students by the medical faculties of the Federal Republic of Germany are systematically ascertained. In regard to the question of the number of international medical students at the medical faculties, only slightly more than a third of the faculties were able to draw on detailed statistics and reported the share of international medical students at an average of 8.8%; with 3.9% EU-foreigners and 4.9% non-EU foreigners.

 Surprisingly, only every third medical faculty registers their international students in detail. A differentiation according to non-mobile foreign students and foreign students would be advantageous in order to provide specific measures for this target group and to inform the targeted population about such offers. Respectively, the systematic registration of international students can be seen as an essential prerequisite for enabling the exchange of information on interventions and support services in the first stages of university education.

Considering the perceived problem areas in regard to the integration of international students, the present study illustrates that the views of the deaneries of the various faculties proved to be very similar. Almost all faculties saw the biggest problem in the fact that the German language skills of international students were seen to be poor at the beginning of their studies, despite having had to demonstrate these as part of the ramifications of their application process in order to gain admittance to university in Germany (through either the TestDaF, eng.:"Test of German as a foreign language" or DSH, eng.: "German language examination for university admission of foreign applicants"), see [https://www.daad.de/deutschland/nach-deutschland/voraussetzungen/de/6221-deutschkenntnisse-nachweisen/ retrieved 18.12.2013]. However, these standardized exams only evaluate general language skills, while specialized medical language competencies are not required to pass. Therefore, it could be important, to promote specific support services teaching medical and scientific-technical language skills in analogy to classes offered to German students interested in clinical traineeships, internships or university education abroad. Successful models, already existing for the graduate area, could strive to teach a combination of general linguistic and expert medical skills [26], [27], [28]. Furthermore, more modern didactic approaches like "lecture-capture” technologies, in which the entire content of a lesson is recorded and provided to students in digital form afterwards [29], appear to be in particularly conducive to international students. Moreover, courses training basic academic writing skills seem to benefit non-native speakers especially [30].

Some faculties also saw serious problems with regard to the integration of international students. In this context language barriers, which are seen as an obstacle to a more effective integration by many non-native speakers, also seem highly relevant [31]. Respectively, tandem or mentoring programs for international students offered in some locations seem useful and promising. Many universities can look back on a long tradition of very popular mentoring and tutor programs for their student body promoting integration, identification with the university and encouraging alumni involvement [32], [33], [34], [35]. Evidently, such programs also enhance the performance of under-represented minorities within the group of medical students [36], possibly resulting from the enhanced intercultural exchange and the thereby facilitated integration of these minorities within the student body. 

Examination situations prove to be a further central difficulty for international students, according to the study deaneries. Students with a non-European immigrant background show significantly lower pass rates in both preclinical semester examinations and in the oral state examination than their fellow German students and also take these exams at significantly later time [23]. In this context, international students may possibly benefit from specific examination preparatory courses offered at a few medical faculties during which the handling of multiple-choice questions is practiced on the basis of exam questions or personal presentation skills are trained by means of simulated oral or practical tests [37]. Further effective test preparation consists of the individual study of case-based medical conditions [38] or in the reduction of test anxiety by learning appropriate coping strategies [39].These training programs could serve as models for especially tailored test preparatory courses appropriated to the specific needs of international students. 

The described problem of the often large intercultural differences could also be met with courses on the field of "medicine and ethno-cultural diversity" in which experienced differences could prove to be subject of valuable discussions. Hence, differences need not be subject of stigmatization, but could rather be integrated into an overall picture. Students of international origin could play a crucial role in the design of such curricula as international studies show that large ethnic and cultural diversity among students and within a faculty has great potential for courses on cultural competence [40]. British and American universities have already been offering such courses as an integral part of the medical curriculum for a long time [41], [42], [43]; in Germany, despite a high proportion of foreign citizens among treated patients, such initiatives still remain to be isolated cases [44]. The implementation of such courses in order to increase intercultural competence is certainly connected with some difficulties; however, if potential pitfalls are known, respective offers can be designed in a manner that greatly benefits both the faculty as well as the individual participants [45].

In result of these findings, given support services varied in the surveyed medical faculties in many ways: There were a number of different offers, both specific student-led tutorials and tandem programs proving to be prominent. This is quite understandable, as tutor-mentoring programs enjoy a high level of acceptance among students in various areas within medical education and training [46], [32]. Moreover, it is assumed that such programs are highly relevant for personal development [47], [48], further career development [49], [50] as well as the development of scientific working methods [51], [50]. Tutorials for international students, designed along the lines of the Peer Assisted Learning principle (PAL), may be helpful in their improved integration and more rapid adaptation to the scientific demands at German universities, as it is known that the teachers of student-led courses are perceived to be more accessible and more familiar with the course and its content [52]; In this context learners show less stress or anxiety [53], improved communication skills [54] and increased confidence in clinical skills [55]. Furthermore, specific medical language courses, aiming at a more experienced handling of German and Latin technical terms, were also offered at several locations. However, the comparison of the number of language support services (36%) with the frequency of international students’ linguistic deficits (83%) reported by the faculties shows room for improvement and highlights an unmet need. Introductory courses, specifically tailored to the needs of international students, were also offered frequently. These have already proven to be beneficial in other contexts (e.g. introductory courses to students at the beginning of the internship year [56]). Specific examination preparatory courses for international students, however, were only offered at every ninth medical school, which is quite surprising in view of the difficulties that international students face in examination situations [23]. The first few months at the beginning of the first semester are seen to be as the most vulnerable phase for international students in a foreign country [57]. Medical schooling is considered to be a labor and learn intensive study subject with comparatively high demands [58]. This makes it all the more difficult for international students to meet the requirements at the beginning of their educational courses. However, in light of the fact that not a single uniform support service was offered by at least half of all faculties, it cannot be said that a firm base of established, standardized interventions currently exists. Each location seemed to pursue its own approach in the integration and support of international students, although peer-to-peer elements were commonly used at many locations. Against this background, the comparative examination of different offers and the identification of the most effective interventions seems to be crucial in order to formulate recommendations for the type and scope of uniform offers of language courses, tutoring programs, introductory courses and test preparatory courses.

The present study shows that many medical faculties offer support services for their international students. Future challenges to be met are, on the one hand, the improved registration of the group of international students and on the other hand the implementation and improvement of targeted tutoring programs, exam preparation, and language courses. An exchange of experiences between German medical faculties on the needs and the effectiveness of interventions for international students seems promising and should be supplemented by supportive research on the objective effectiveness of interventions for international students. Here, future research in the field of test preparation and peer-teaching programs can build on prior experiences of the evaluation and the effectiveness of such interventions from other areas of medicine (exam preparation [59], Peer Assisted Learning (PAL) [60]).

Limitations of this study are to be seen in the fact that only employees of the respective academic deaneries, however, not the affected students themselves were heard. Though a survey of the international students themselves would be costly, the analysis of their experiences and the respective recommendations of this group could prove to be purposeful and beneficial. Further research should seek to clarify the expectations, needs and experiences of this specific group of international students. A further limitation to be noted is the fact that the differentiation of EU and non-EU foreigners as well as a categorization of mobile and non-mobile students would be very beneficial. However, a precise definition and registration of the corresponding students is extremely difficult, resulting in the fact that there is usually almost no useful information available. A more detailed differentiation of personal data would certainly be helpful for the development of targeted offers in future.

## Acknowledgement

We would like to thank Anna Cranz for excellent proof-reading.

## Funding

Funded under the program »Innovation Fund Education Research» t the Universities of the Ministry of Science, Research and Arts Baden-Württemberg: "**Assurance of academic success in high-risk groups - tudy on improving the intercultural communication**" Grant no.: 42-04HV.MED(12)/29/8

## Competing interests

The authors declare that they have no competing interests.

## Figures and Tables

**Table 1:**
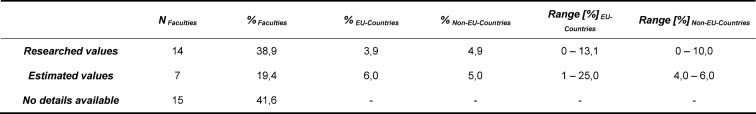
Descriptive representation of data of the proportion of international medical students, differentiated according to EU and non-EU immigrant background; Data in n= absolute number, %- share and range of percentages

**Table 2:**
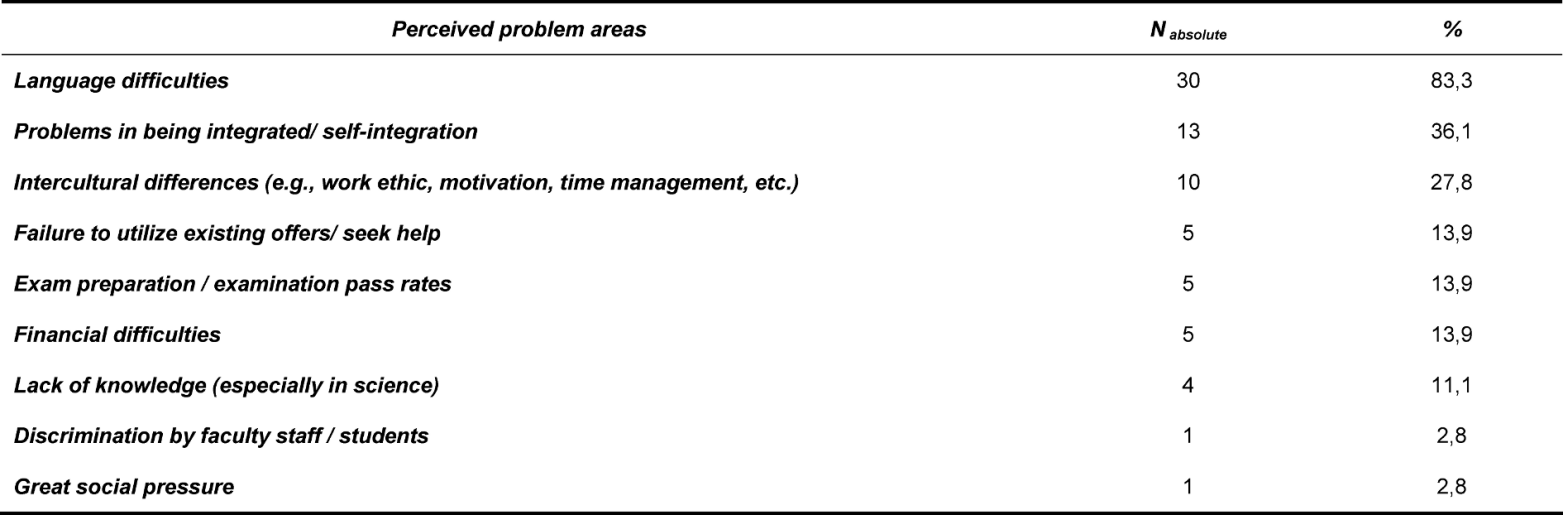
Descriptive representation of the biggest perceived problem areas of international medical students (multiple answers possible); Data in n=absolute number and %- share

**Table 3:**
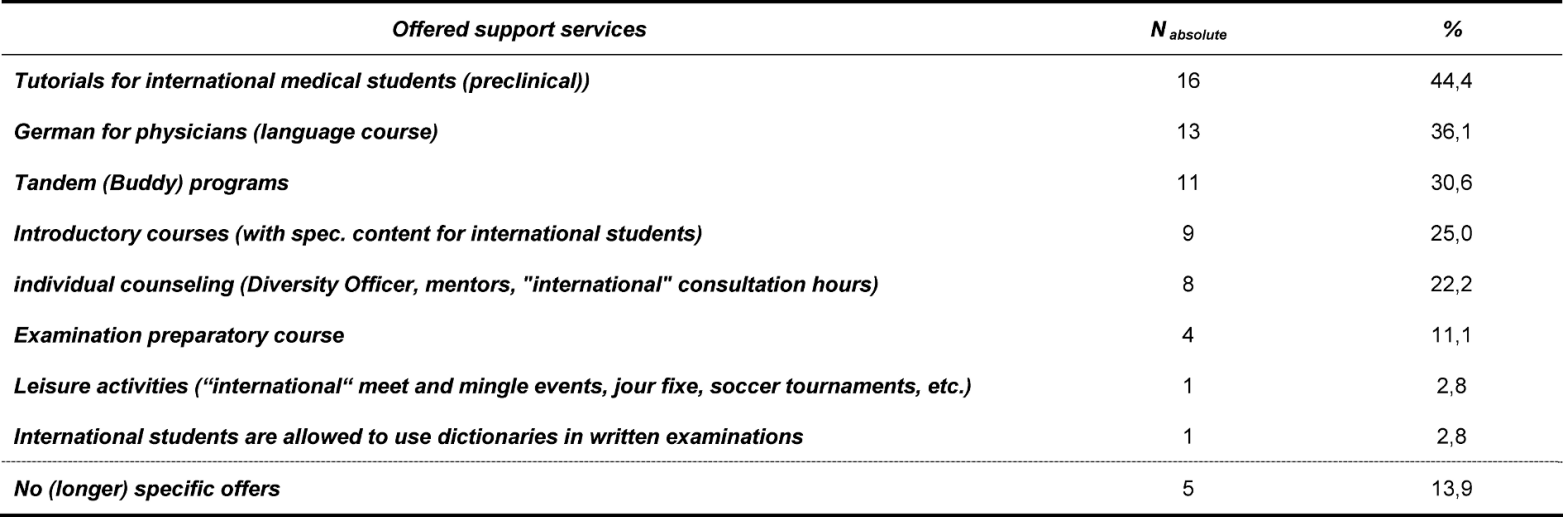
Descriptive representation of specific programs for international medical students; Data in n=absolute number and %- share

**Table 4:**
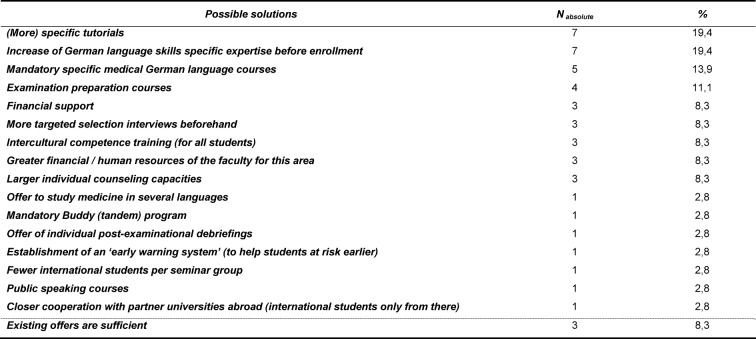
Descriptive representation of the addressed possible solutions for improving the conditions of international medical students (multiple answers possible); Data in n=absolute number and %- share
